# Red Dress Day in Croatia: stroke prevention based on sex differences

**DOI:** 10.3325/cmj.2020.61.5

**Published:** 2020-02

**Authors:** Arijana Lovrenčić-Huzjan, Zdravka Poljaković

**Affiliations:** 1 Sestre Milosrdnice University Hospital Center, Zagreb, Croatia *arijana.huzjan@gmail.com*; 2 University Hospital Center Zagreb, Zagreb, Croatia

Stroke is a major cause of death and disability worldwide. In the United States, it ranks fifth among leading causes of death in men and fourth in women (1). In Croatia, cerebrovascular diseases were the second main cause of mortality in 2018, leading to fatal outcome in 13.3% of women and 9.9% of men. Stroke burden is further increased by the fact that one third of the patients remains disabled.

Women have higher lifetime stroke risk than men due to their longer lifespan. They also have a higher stroke burden in terms of institutionalizations after a stroke, which are often necessary in widows or women who live alone. The burden in women may be still higher owing to a greater prevalence of mood disorders, depression, decreased motility, and lower quality of life (2).

## Stroke in women – strong evidence of sex influence on clinical presentation and outcome

The first vascular event in men is coronary heart disease, whereas in women it is stroke. Despite higher disease burden in women, clinical research on stroke was mostly focused on assessing treatment outcomes rather than exploring the impact of gender. However, clinical experience showed that the actual stroke patient was not a typical patient enrolled in clinical trials, sparking research interest in gender differences in stroke.

Sex strongly affects the disease development. Women are at an increased risk of venous sinus thrombosis due to hypercoagulability associated with pregnancy and postpartum period. Hypertension in pregnancy increases the risk for preeclampsia/eclampsia and pregnancy-associated stroke, and eclampsia further increases stroke risk within 3 years after pregnancy.

Among traditional risk factors that have a higher impact on stroke in women are hypertension, atrial fibrillation, and diabetes mellitus. Women are also disproportionately affected by non-vascular risk factors, such as psychosocial stress and depression. Another factor with a negative impact on stroke outcome was found to be frailty (3). Frailty may be defined as a clinical syndrome characterized by slow walking speed, weakness assessed by handgrip, self-reported low physical activity level, exhaustion, and unintended weight loss (4). Furthermore, migraine proved to be a risk factor for stroke in women but not in men (5), with exponentially increased risk in women who smoke or use oral contraceptives (5). Women also more frequently suffer from intracranial aneurysms, as well as some rare types of stroke, such as reversible cerebral vasoconstrictor syndrome, posterior reversible encephalopathy, and spontaneous cervical artery dissection. While men are more often susceptible to atherosclerotic, macroangiopathic disease, women are more susceptible to less recognized vasculopathies, such as fibromuscular dysplasia, which may contribute to a worse outcome.

Atypical stroke presentation in women, in terms of cortical associated symptoms or impaired consciousness, may hinder the diagnosis of stroke in the emergency department. Furthermore, women present to the emergency department later than men, most often because they live alone, leading to a delayed active stroke treatment (6).

Therefore, the American Heart Association/American Stroke Association in 2014 published the Guidelines for the Prevention of Stroke in Women (7). These guidelines emphasize the control of stroke risk factors unique to female sex and point to the risk factors that are more prevalent in women.

**Figure 1:**
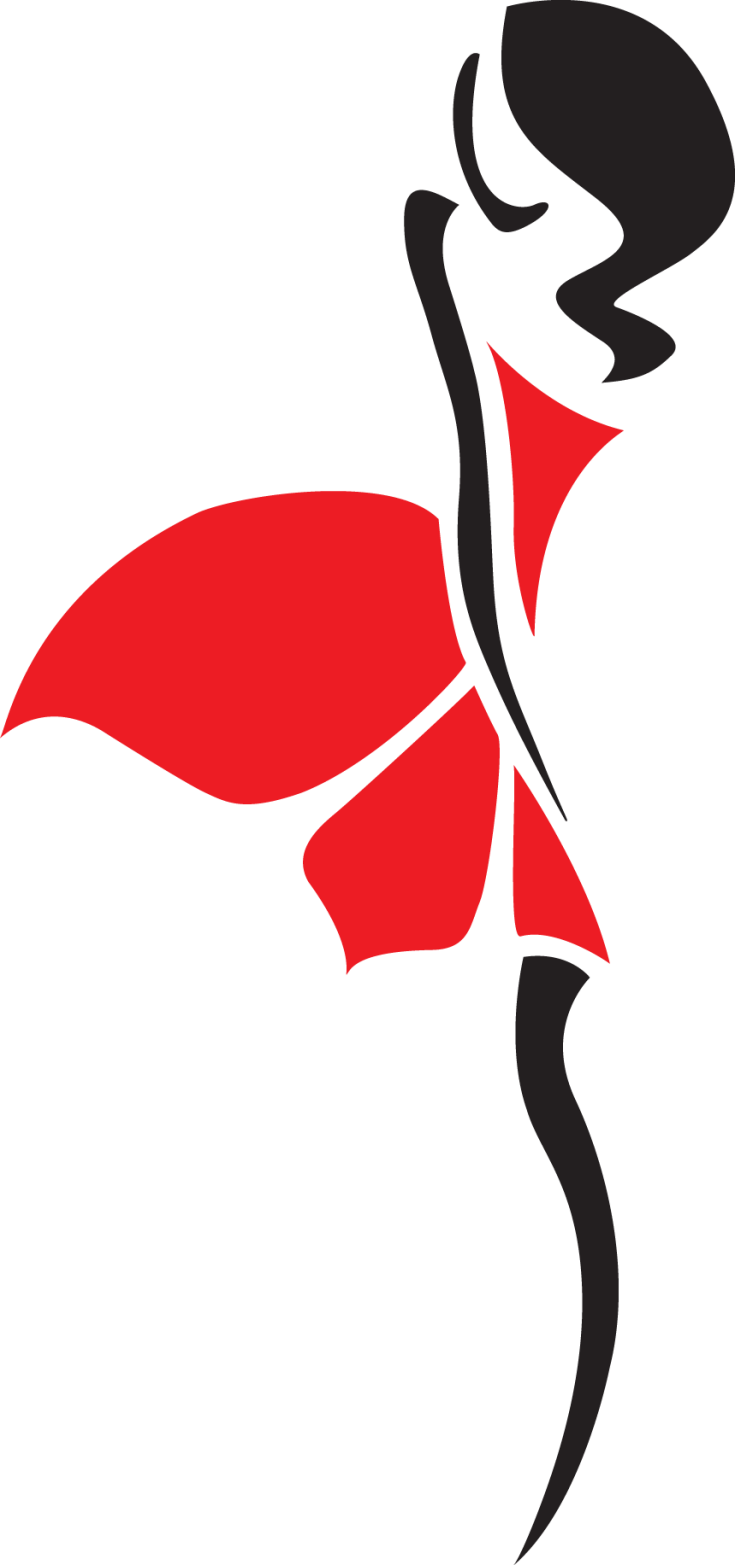
The logo of Red Dress Day in Croatia (designed by Petra Huzjan).

Since most strokes are preventable, publicity campaigns are aimed at preventing the risk factors. One such campaign, National Wear Red Day, was launched in the United States with the aim to raise the awareness of vascular risk factors. We have organized a similar campaign in Croatia, called Red Dress Day (Figure 1), which was aimed to highlight the specificities of female stroke risks. During the campaign, brave women who suffered stroke shared their experiences in the media. At the final ceremony on February 1, 2019, these women were celebrities for the night, walking the runway in red dresses by Croatian designers.

## Clinical trials – do they address accurately the impact of sex on disease

As women are under-represented in active treatment trials, trial results can be applied mainly to men. Furthermore, older women are often “atypical stroke patients,” with more comorbidities, which prevents their inclusion in clinical trials. The treatment results of such patients, therefore, differ from the published results. The under-representation of women in major randomized clinical trials raised doubt about the results on safety and efficacy of the drugs (7) and has been suggested as a reason why women had more drug-related adverse events (8).

In 2018, the Secretary-General of United Nations General Assembly highlighted this issue by calling for gender-based approaches for the prevention and control of non-communicable diseases. These approaches should include organizing focused stroke awareness campaigns for women; conducting research on the obstacles to regular screening for stroke risk factors, seeking acute care, and receiving post stroke care in women; enhancing the inclusion of women >80 years in stroke randomized controlled trials; and halting male enrollment in randomized controlled trials after achieving 50% of the sample size (9,10).

## Focused and gender-based stroke prevention initiatives

Despite the progress of personalized medicine, we are still prone to overlook the impact of sex on different pathologies. Overall, gender and sex differences in every aspect of stroke, from epidemiology to treatment, demand further research, and lack of awareness of a worse stroke outcome in women demands focused campaigns. Red Dress Day, initiated in Croatia in 2019, is an event promoting women’s health and stroke prevention by education and recognition of gender-specific symptomatology. We are proud to be part of worldwide preventive actions focused on those who are most affected by the disease.

## References

[1] National Center for Health Statistics. Health, United States, 2011. Hyattsville, MD: US Department of Health and Human Services, Centers for Disease Control and Prevention, National Center for Health Statistics; 2018. Available from: https://www.cdc.gov/nchs/data/hus/2018/006.pdf*.* Accessed: February 25, 2020.

[2] Bushnell CD Reeves MJ Zhao X Pan W Prvu-Bettger J Zimmer Let alSex differences in quality of life after ischemic stroke. Neurology 2014 82 922 31 10.1212/WNL.0000000000000208 24510493 PMC4211921

[3] Fried LP, Tangen CM, Walston J, Newman AB, Hirsch C, Gottdiener J (2001). Frailty in older adults: evidence for a phenotype.. J Gerontol A Biol Sci Med Sci.

[4] Singh M, Stewart R, White H (2014). Importance of frailty in patients with cardiovascular disease.. Eur Heart J.

[5] Sheikh HU, Pavlovic J, Loder E, Burch R (2018). Risk of stroke associated with use of estrogen containing oral contraceptives in women with migraine.. Headache.

[6] (2014). Sex differences and stroke prevention.. Lancet Neurol.

[7] Bushnell CD, McCullough LD, Awad IA, Chireau MW, Fedder WN, Furieet KL (2014). Guidelines for the prevention of stroke in women.. Stroke.

[8] Tsivgoulis G, Katsanos AH, Caso V (2017). Under-representation of women in stroke randomized controlled trials: inadvertent selection bias leading to suboptimal conclusions.. Ther Adv Neurol Disord.

[9] United States Government Accounting Office (GAO). Drug Safety: Most Drugs Withdrawn in Recent Years Had Greater Health Risks For Women. GAO-01-286R, 2001. Available from: www.gao.gov/new.items/d01286r.pdf. Accessed: February 25, 2020.

[10] Baschieri F, Acciarresi M, Caso V (2018). Gender-based approaches for the prevention and control of noncommunicable diseases. Keep an eye on stroke.. Stroke.

